# Kinase activity profiling in renal cell carcinoma, benign renal tissue and in response to four different tyrosine kinase inhibitors

**DOI:** 10.18632/oncotarget.28257

**Published:** 2022-08-04

**Authors:** Andliena Tahiri, Katarina Puco, Faris Naji, Vessela N. Kristensen, Glenny Cecilie Alfsen, Lorant Farkas, Frode S. Nilsen, Stig Müller, Jan Oldenburg, Jürgen Geisler

**Affiliations:** ^1^Institute of Clinical Medicine, University of Oslo, Oslo, Norway; ^2^Department of Medical Genetics, Oslo University Hospital, Ullevål, Norway; ^3^Department of Clinical Molecular Biology (EpiGen), Akershus University Hospital, Lørenskog, Norway; ^4^Department of Oncology, Akershus University Hospital, Lørenskog, Norway; ^5^Pamgene International BV, ‘s-Hertogenbosch, The Netherlands; ^6^Department of Pathology, Akershus University Hospital, Lørenskog, Norway; ^7^Department of Urology, Akershus University Hospital, Lørenskog, Norway; ^*^These authors contributed equally to this work

**Keywords:** kidney cancer, kinase activity, tyrosine kinase inhibitors, renal cell carcinoma, tyrosine kinase

## Abstract

Kinase activity is frequently altered in renal cell carcinoma (RCC), and tyrosine kinase inhibitors (TKIs) are part of the standard treatment strategy in patients with metastatic disease. However, there are still no established biomarkers to predict clinical benefits of a specific TKI. Here, we performed protein tyrosine kinase (PTK) profiling using PamChip^®^ technology. The aim of this study was to identify differences in PTK activity between normal and malignant kidney tissue obtained from the same patient, and to investigate the inhibitory effects of TKIs frequently used in the clinics: sunitinib, pazopanib, cabozantinib and tivozanib. Briefly, our results showed that 36 kinase substrates differs (FDR < 0.05) between normal and cancer kidney tissue, where members of the Src family kinases and the phosphoinositide-3-kinase (PI3K) pathway exhibit high activity in renal cancer. Furthermore, *ex vivo* treatment of clear cell RCC with TKIs revealed that pathways such as Rap1, Ras and PI3K pathways were strongly inhibited, whereas the neurotrophin pathway had increased activity upon TKI addition. In our assay, tivozanib and cabozantinib exhibited greater inhibitory effects on PTK activity compared to sunitinib and pazopanib, implying they might be better suitable as TKIs for selected RCC patients.

## INTRODUCTION

Renal cell carcinoma (RCC) is the third most common type of urinary cancer in the world, and the sixth most common cancer type in Europe. Approximately 403 000 new cases and 175 000 deaths were registered worldwide in 2018 [1, 2]. Although most patients have localized disease at presentation, 20–40% experience either local or distant relapse, requiring systemic treatment. In addition, approximately 25% of all kidney cancer patients are presented with metastatic disease already at the time of diagnosis [3].

RCC is a malignancy comprising different histological subtypes with distinctive genetic and molecular alterations [4]. The three major histologic subtypes are clear cell renal carcinoma (ccRCC), which accounts for ~75% cases, papillary renal cell carcinoma (pRCC), which accounts for 15–20%, and chromophobe renal cell carcinoma (ChRCC), representing ~5% of all RCC cases. Principally, metastatic RCC (mRCC) is incurable and affected patients require systemic treatment which only confers palliative management.

The tumor development and cellular proliferation that take place in RCC may be accredited to changes in intracellular activity, involving many growth factors and growth factor receptors. The proangiogenic growth factors such as vascular endothelial growth factor (VEGF), VEGF receptor (VEGFR), platelet derived growth factor receptor (PDGFR), and basic fibroblast growth factor (bFGF) are specifically overexpressed in RCC, which indicates that the tumor is in a highly vascular state [5]. As a result, a range of therapies have been developed targeting these growth factor receptors via the phosphatidylinositol-3-kinase (PI3K)/Akt and the mammalian target of rapamycin (mTOR) signaling pathways, including TKIs (sorafenib, sunitinib, pazopanib), VEGF antibodies (bevacizumab) and mTOR inhibitors (everolimus, temsirolimus) [6–8]. In addition, immune checkpoint inhibitors (pembrolizumab, nivolumab, avelumab and ipilimumab) are available for systemic treatment of mRCC, with promising results [9]. Combination treatment with checkpoint-inhibitors and TKIs are the new standard treatment in the first-line setting, as well as the combination of different checkpoint inhibitors (immunotherapy doublet) for some subgroups of patients. Except for the immunotherapy doublet, virtually all lines of systemic RCC treatment recommended by clinical practice guidelines consist of TKIs. Despite a number of new treatment options improving RCC patients’ disease control rates and survival, the lack of useful biomarkers remains a major clinical concern. Cancer progression during TKI therapy in mRCC is inevitable and subsequent treatment lines with TKIs usually yield ever-shortening progression free survival (PFS) as RCC clones resistant to the applied TKIs are positively selected, rendering the RCC increasingly therapy resistant. Of note, both TKI-induced benefits and toxicity display remarkable inter-individual variation. Where some patients respond well for several months and even years from one TKI without noteworthy toxicity, others may have lower quality of life due to fatigue, diarrhea, hypertension, sore hands and feet, and oropharyngeal ulcers without affecting their progression to mRCC. Clinical parameters and predefined clinical risk groups are established for first line treatment [10, 11], but do not represent a substitute for tumor- and patient- specific biomarkers predicting the likelihood of clinical benefit for distinct TKIs.

Thus, the aim of this study was to expand the understanding of the tyrosine kinome of human kidney cancer before and during TKI therapy to identify possible biomarkers of clinical relevance that might help to select the optimal patient groups for therapeutic interventions involving targeting compounds. In the present study, we focused primarily on the overall tyrosine kinase activity patterns in RCC and the effects of *ex vivo* TKIs. At the same time, we were looking for particularly important pathways and potential novel therapeutic targets.

## RESULTS

### Tyrosine kinase activity profiles in cancer and normal kidney tissue

**Figure 1:**
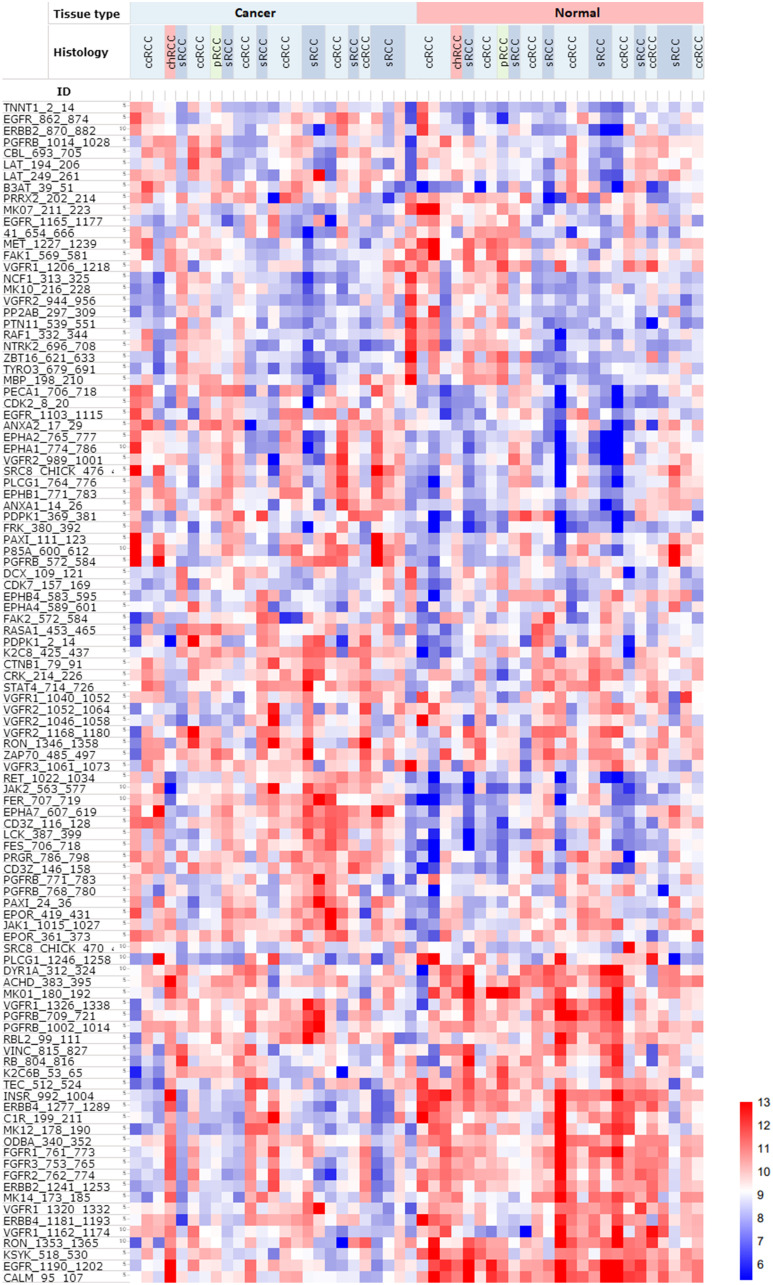
Heatmap of PTK phosphorylation profiles of 25 RCC patients, including malignant and matched normal tissue from the same patient (*n* = 50). There is a clear separation of normal and cancer kidney tissue, but no significant differences amongst the different histological types. Red indicate higher phosphorylation, whereas blue indicate lower phosphorylation of PTK. Samples are displayed on the horizontal axis, whereas kinase substrates are displayed on the vertical axis. Abbreviations: ccRCC: clear cell carcinoma; chRCC: chromophobe renal cell carcinoma; pRCC: papillary renal cell carcinoma; sRCC: renal cell carcinoma with sarcomatoid features.

Unsupervised heatmap analysis of kinase substrate phosphorylation profiles showed distinct differences in phosphorylation patterns between normal and cancerous kidney tissue using log2-transformed values (Figure 1). We identified 36 kinase substrates that had significantly different phosphorylation profiles (False discovery rate (FDR) < 0.05) in normal and cancer tissue (Table 1). Twenty substrates showed higher kinase activity in cancer, whereas 16 substrates showed significantly lower kinase activity in cancer compared to normal kidney tissue (FDR < 0.05). Through pathway analysis we identified that most kinase substrates that exhibited high kinase activity were part of the PI3K-akt pathway (*p*.value = 3.1E-3, Benjamini = 9.0E-2), whereas kinase substrates within the Rap1 signaling pathway (*p*.value = 1.7E-9, Benjamini= 1.9E-7) showed lower kinase activity in cancer, and higher in normal kidney tissue. We could not identify any significant differences in phosphorylation profiles between different histological groups, or other clinical parameters (age, gender, risk profile etc.).

**Table 1:** Significant kinase substrates (FDR < 0.05) between normal and RCC tissue

Substrate ID	Description	*p*.value	FDR	Delta
CD3Z_116_128	T-cell surface glycoprotein CD3 zeta chain precursor	1,9E-04	1,5E-03	0,296
CD3Z_146_158	T-cell surface glycoprotein CD3 zeta chain precursor	4,0E-04	2,6E-03	0,278
ANXA2_17_29	Annexin A2	1,2E-03	6,6E-03	0,255
EPHA1_774_786	Ephrin type-A receptor 1 precursor	1,0E-02	3,4E-02	0,241
EPHA7_607_619	Ephrin type-A receptor 7 precursor	3,6E-04	2,5E-03	0,232
FES_706_718	Proto-oncogene tyrosine-protein kinase Fes/Fps	6,1E-05	9,0E-04	0,219
JAK2_563_577	Tyrosine-protein kinase JAK2	5,8E-05	9,0E-04	0,209
EPHA2_765_777	Ephrin type-A receptor 2 precursor	1,6E-02	4,7E-02	0,203
CDK2_8_20	Cell division protein kinase 2	1,8E-03	8,3E-03	0,200
RET_1022_1034	Proto-oncogene tyrosine-protein kinase receptor ret precursor	1,4E-04	1,4E-03	0,182
PECA1_706_718	Platelet endothelial cell adhesion molecule precursor	2,9E-03	1,2E-02	0,178
FER_707_719	Proto-oncogene tyrosine-protein kinase FER	1,6E-04	1,4E-03	0,174
PDPK1_369_381	3-phosphoinositide-dependent protein kinase 1	8,6E-03	2,9E-02	0,174
LCK_387_399	Proto-oncogene tyrosine-protein kinase LCK	1,2E-03	6,6E-03	0,146
K2C8_425_437	Keratin, type II cytoskeletal 8	2,2E-03	9,6E-03	0,143
FRK_380_392	Tyrosine-protein kinase FRK	7,0E-03	2,5E-02	0,131
ANXA1_14_26	Annexin A1	1,4E-02	4,3E-02	0,119
EPHB1_771_783	Ephrin type-B receptor 1 precursor	3,2E-03	1,2E-02	0,113
B3AT_39_51	Band 3 anion transport protein	1,3E-02	3,9E-02	0,106
EPOR_361_373	Erythropoietin receptor precursor	1,1E-02	3,5E-02	0,094
TEC_512_524	Tyrosine-protein kinase Tec	8,7E-03	2,9E-02	−0,076
MK14_173_185	Mitogen-activated protein kinase 14	1,6E-03	8,0E-03	−0,153
RON_1353_1365	Macrophage-stimulating protein receptor precursor	1,5E-03	7,7E-03	−0,163
MK01_180_192	Mitogen-activated protein kinase 1	3,2E-03	1,2E-02	−0,187
MK12_178_190	Mitogen-activated protein kinase 12	1,4E-04	1,4E-03	−0,192
ERBB2_1241_1253	Receptor tyrosine-protein kinase erbB-2 precursor	3,0E-04	2,2E-03	−0,226
FGFR3_753_765	Fibroblast growth factor receptor 3 precursor	8,6E-05	1,1E-03	−0,247
ODBA_340_352	2-oxoisovalerate dehydrogenase subunit alpha, mitochondrial precursor	8,8E-04	5,1E-03	−0,252
KSYK_518_530	Tyrosine-protein kinase SYK	6,2E-06	1,3E-04	−0,253
C1R_199_211	Complement C1r subcomponent precursor	3,4E-03	1,2E-02	−0,261
FGFR2_762_774	Fibroblast growth factor receptor 2 precursor	1,2E-04	1,4E-03	−0,291
FGFR1_761_773	Basic fibroblast growth factor receptor 1 precursor	7,8E-04	4,8E-03	−0,308
ERBB4_1277_1289	Receptor tyrosine-protein kinase erbB-4 precursor	1,5E-06	4,0E-05	−0,520
EGFR_1190_1202	Epidermal growth factor receptor precursor	1,2E-06	4,0E-05	−0,521
CALM_93_105	Calmodulin	9,5E-08	4,9E-06	−0,573
INSR_992_1004	Insulin receptor precursor	8,2E-08	4,9E-06	−0,676

Abbreviation: ^*^FDR: False discovery rate; Delta: differences between normal and cancer tissue. Positive value indicates higher expression in cancer, whereas negative value indicates lower expression in cancer.

### Upstream kinases that might be responsible for differences observed between normal and cancerous tissue

**Figure 2:**
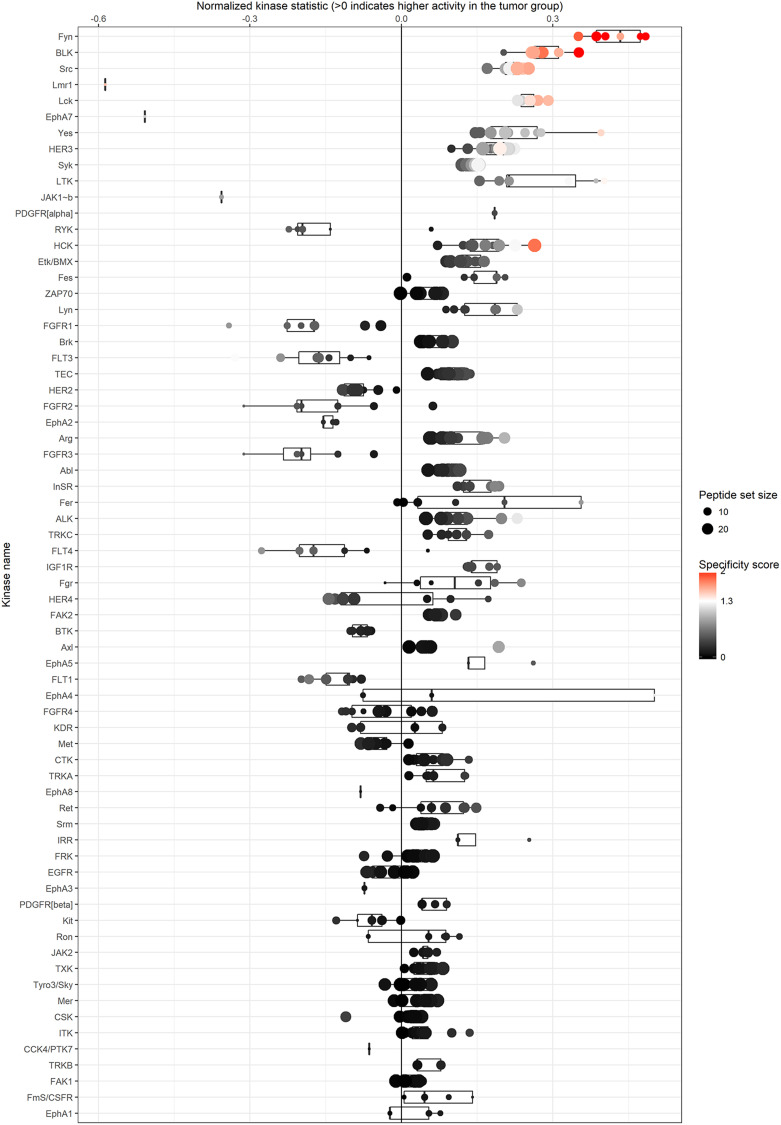
Upstream kinase analysis identifies kinases that might be responsible for the differences in phosphorylation profiles between normal and cancer tissue. Kinases at the top are the ones that are most likely to be involved compared to the ones at the bottom of the list. Src-kinase family members seems to be dominating at the top.

In order to identify the kinases potentially responsible for the observed differences in kinase activity between normal and cancerous tissue, we performed upstream kinase analysis. Through this analysis we identified a list of predicted kinases, with several of them showing higher kinase activity in cancerous compared to normal kidney tissue (Figure 2). Amongst them were members of the Src family kinases such as Fyn, BLK, Src, LCK, Yes, HCK, Lyn and Fgr. Some kinases identified had lower kinase activity in cancer compared to normal tissue, amongst them Lmr1, EPHA7 and JAK1.

### Tyrosine kinase profiles upon *ex vivo* inhibition with TKIs and analysis of affected pathways

**Figure 3:**
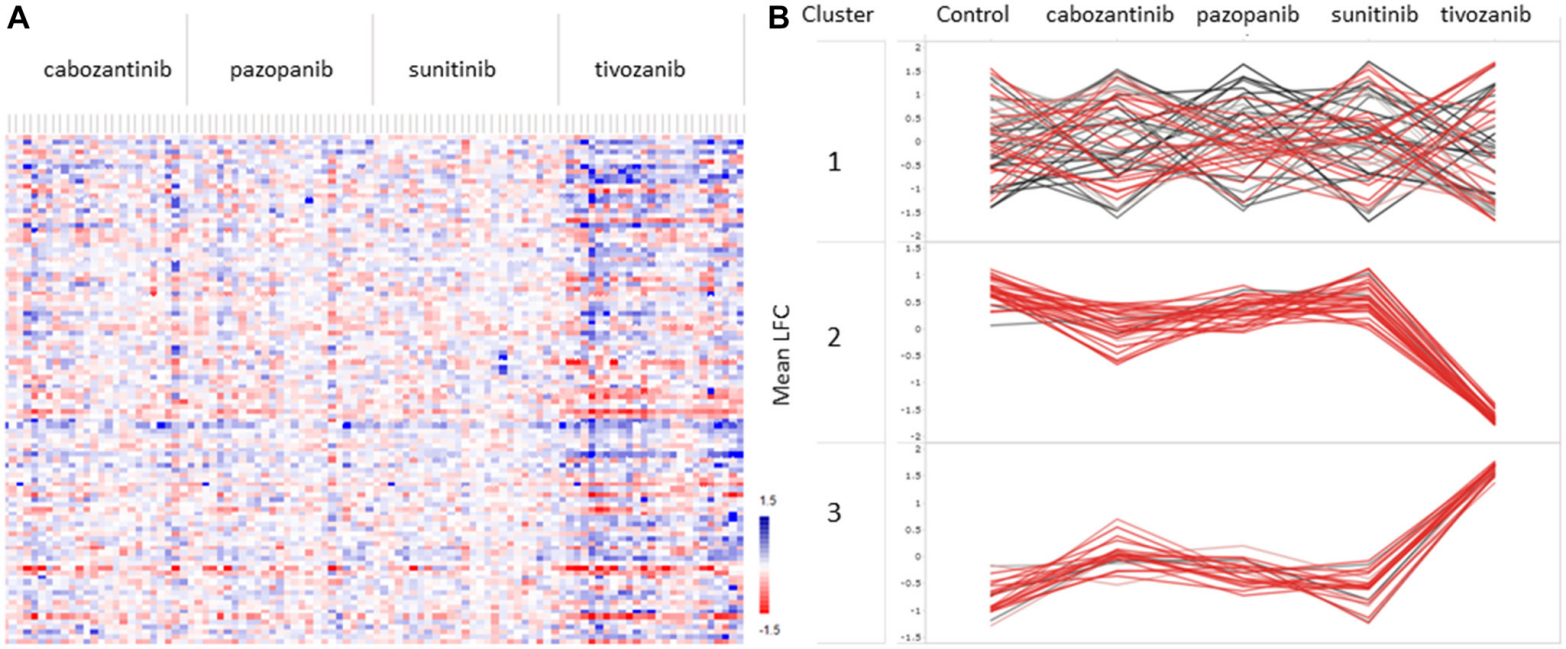
A Mean log fold change (LFC) values between RCC tissue without treatment (control) and with *ex vivo* treatment with four different tyrosine kinase inhibitors (TKIs): sunitinib, pazopanib, cabozantinib, and tivozanib. (**A**) Heatmap of mean LFC values of each kinase substrate in all samples treated with different TKIs. Red indicates positive LFC between control and TKI, whereas blue indicates negative LFC between control and TKI. (**B**) Kinase substrates were clustered in three groups, cluster 1, 2 and 3, based on PTK activity profiles in treated versus untreated (control) RCC samples. Cluster 2 and cluster 3 showed similar trends upon treatment with TKIs, whereas substrates in cluster 1 were affected differently by all TKIs in different samples. Values indicate the mean LFC between treated and untreated samples.

The inhibition profiles obtained during *ex-vivo* exposure of cancer tissue lysates to four different TKIs (pazopanib, tivozanib, sunitinib and cabozantinib) showed altered kinase activity (Figure 3A). The heatmap shows the log fold change (LFC) values between control and treated RCC. Due to the low number of samples of pRCC and chRCC, we could not get a clear indication of the differences between histological kidney cancer types, and they were therefore excluded from the analysis with TKIs. From our results, tivozanib clearly exhibited stronger inhibitory potential compared to the other TKIs (Supplementary File 1).

Further, we identified three distinct clusters of kinase substrates that were differently affected by the TKIs (Figure 3B, Supplementary File 2). Cluster 1 contained 54 kinase substrates that were differently affected by all four TKIs. Cluster 2 consisted of 29 kinase substrates showing reduced kinase activity upon treatment with all four of the TKIs. Pazopanib and sunitinib exhibited little inhibitory effects on most samples, whereas cabozantinib and tivozanib clearly showed inhibition of kinase substrates in cluster 2, exhibiting greater potency. Interestingly, 21 kinase substrates in cluster 3 showed increased kinase activity upon treatment with TKIs, with tivozanib showing greater potency in activating these kinases. Supplementary File 3 contains information on *P*-values and LFC values of each kinase substrate upon treatment with the different TKIs, and the cluster to which each kinase substrate belongs.

Pathway analysis was performed using UNIPROT accession number of each kinase substrate in cluster 2 and cluster 3 in order to identify the pathways affected by the TKIs (Supplementary File 2). Here, the results show that the PI3K pathway is overrepresented, especially in cluster 2. Kinase substrates encoding for endothelial growth factor receptor (EGFR), Src substrate protein p85 (p80) and VEGFR1 and VEGFR2 are both in cluster 2 and cluster 3, but the phosphorylation sites of these kinases are different in the two clusters. In addition to PI3K, kinases within the Rap1 and Ras signaling pathways are highly affected by the TKIs in cluster 2, whereas kinases within the neurotrophin and Ras signaling pathway are activated by the TKIs (Table 2).

**Table 2:** Selected kinase substrates in cluster 2 and 3 involved in different pathways

Kinase substrate ID	UNIPROT Accession	Pathway	Cluster
EGFR_1190_1202	P00533	Rap1 /Ras/PI3K	2
FGFR1_761_773	P11362	Rap1 /Ras/PI3K	
FGFR3_753_765	P22607	Rap1 /Ras/PI3K	
PGFRB_1002_1014	P09619	Rap1 /Ras/PI3K	
PGFRB_709_721	P09619	Rap1 /Ras/PI3K	
VGFR1_1326_1338	P17948	Rap1/Ras/PI3K	
CALM_95_107	P62158	Rap1 / Ras	
VGFR2_1052_1064	P35968	Ras /PI3K	
EPOR_361_373	P19235	PI3K	
JAK1_1015_1027	P23458	PI3K	
RBL2_99_111	Q08999	PI3K	
CRK_214_226	P46108	Rap1	
CTNB1_79_91	P35222	Rap1	
VGFR2_1168_1180	P35968	Rap1	
RASA1_453_465	P20936	Ras	
ZAP70_485_497	P43403	Ras	
MK07_211_223	Q13164	Neurotrophin	3
MK12_178_190	P53778	Neurotrophin	
NTRK2_696_708	Q16620	Neurotrophin	
RAF1_332_344	P04049	Neurotrophin / Ras	
MK10_216_228	P53779	Neurotrophin /Ras	
PLCG1_1246_1258	P19174	Neurotrophin /Ras	
PTN11_539_551	Q06124	Neurotrophin /Ras	
EGFR_1165_1177	P00533	Ras	
LAT_194_206	O43561	Ras	
VGFR1_1206_1218	P17948	Ras	
VGFR2_1046_1058	P35968	Ras	
VGFR2_944_956	P35968	Ras	

## DISCUSSION

We identified significant differences in the overall PTK activity when comparing normal and cancer kidney tissue samples from patients diagnosed with RCC. Especially kinases within the PI3K pathway had higher activity in cancer compared to normal kidney tissue, but the PI3K pathway was also most affected by the TKIs in our *in-vitro* experiments. On the contrary, kinases within the Rap1 pathway were noted to be less active in cancer and higher in normal kidney tissue, but after *ex vivo* TKI treatment of cancer tissue, the Rap1 signaling pathway seemed to be the most affected. This indicates that these pathways play an important role in RCC patients and treatment response.

A recent study by Anderson et al. assessed the kinase activity profiles of ccRCC patients, and identified a number of kinase substrates different between normal and cancer kidney tissue [12]. However, although they had a smaller dataset of matched normal and cancerous tissue samples (*n* = 12), and their findings predominantly included serine/threonine kinases (STKs), our findings support that the kinase substrate encoding for the erythropoietin-producing hepatoma B1 (EPHB1) protein is higher phosphorylated in cancer tissue compared to normal tissue (Table 3). EPH proteins are involved in cell processes such as cell growth and differentiation, and have long been suggested as potential targets in cancer treatment as overexpression of EPH proteins have been found in several types of cancer, including kidney cancer [13]. High EPH-ephrin signaling has been shown to be involved in the pathogenesis and progression of ccRCC, with worse prognosis associated with higher expression of EPHA1, EPHA2 and EPHA7 [14, 15]. In our dataset, in addition to EPHB1, we also identified EPHA1, EPHA2 and EPHA7 that were highly phosphorylated in cancer compared to normal tissue, making these proteins as potential therapeutic targets. In addition, upstream kinase analysis confirmed by both our study and that of Anderson et al. [12], that members of the Src family kinase proteins are significantly different in normal and kidney cancer tissue, including Fyn, Src and Lyn, exhibiting higher kinase activity levels in cancer. Research involving RCC has shown that Src is the most highly expressed gene, followed by Lyn, Hck, Fgr and Fyn [16], which fits well with our observations. Interestingly, the over-expression of Src family members has also been observed to have important roles in other types of malignancy, including prostate, breast, colon, and lung cancer [17–20]. In breast cancer especially, Src overexpression has been correlated to poor survival [19] and resistance to therapy [21]. Furthermore, interaction between EPHB1 and Src has been shown to activate Ras/Raf/MAPK pathway [22], and is concordant with the results we have obtained throughout our analysis. Previous research involving analysis on several “omic” levels on a large number of ccRCC patients revealed that several genes within the PI3K/AKT pathway were often mutated (28% of cases) [23], suggesting that kinases within this pathway are important potential therapeutic targets. Our study had a limited number of patients with different histological subtypes, and therefore our findings did not show major differences between different subtypes, i.e. pRCC versus ccRCC, nor did we find any significant associations between PTK profiles and clinical parameters. For future research, larger number of patients should be included, including different histological types.

**Table 3:** Patient characteristics

Characteristics	Number (*n* =)
**Gender**	
Female	9
Male	16
**Age group**	
Median (range)	65 (43–79)
**Histology**	
Clear cell	23
Papillary	1
Chromophobe	1
**IDMC/ MSKCC risk group**	
Good	10
Intermediate	11
Poor	1
Non-metastatic disease	3

Abbreviations: ^*^IDMC: International Metastatic Renal Cell Carcinoma Database Consortium; MSKCC: Memorial Sloan Kettering Cancer Center.

In this study, we also explored the effects of four different TKIs on kinase activity in RCC that are commonly used in clinical settings, such as pazopanib, sunitinib, cabozantinib and tivozanib. Our analysis showed that both cabozantinib and tivozanib exhibited greater potency with regards to decreasing phosphorylation levels, but they also showed greater activation of certain kinases. Sunitinib and pazopanib on the other hand, did not show the same potency in decreasing phosphorylation levels in RCC samples. Based on our results, tivozanib exhibited the best inhibitory effect on PTKs. Tivozanib is a VEGF-inhibitor that has been extensively studied in the context of solid tumors and in advanced RCC through preclinical data and clinical trials but has only recently (late 2017) been approved as therapy for mRCC patients [24, 25]. Previous studies have shown that tivozanib is effective for patients that have previously received TKI treatment and is superior to other TKIs as it prolongs response of mRCC patients substantially [26]. For future studies, there should be research studying whether tivozanib given as a first line drug might improve lifespan of RCC patients, as it shows greater inhibitory potential, although a very low concentration of the drug was used for the *in vitro* experiments.

Furthermore, we identified two clusters of kinase substrates that were either negatively affected (cluster 2), or positively affected by all four TKIs (cluster 3). Our findings suggest that while some kinases are targeted specifically by the TKIs and are therefore inhibited, others in the same pathway will compensate and increase their activity. We have observed a similar phenomenon in our previous study with malignant melanoma and treatment with *ex vivo* BRAF-inhibitor, with some kinases being inhibited, whereas others being activated upon TKI treatment [27], revealing the complexity of the use of TKIs in the clinics. Furthermore, we identified that kinases within the Rap1 and Ras pathway were significantly affected by TKIs. Ras and Rap1, play critical roles in regulating T- cell proliferative responses. Ras plays an essential role in transmitting signals from the T-cell receptor (TCR) to activation of the Raf-1/ERK signaling cascade, which is required for T- cell proliferation, IL-2 production, and thymic maturation [28]. Rap1, is also activated in T-lymphocytes following TCR stimulation, and is a known suppressor of Ras-dependent transformation [29]. Our study also showed that kinases within the neurotrophin and Ras signaling pathway were activated upon TKI treatment. Activation of the neurotrophin pathway can stimulate the Ras, PI3K, phospholipase C-γ1 signaling pathways controlled through these proteins, including the MAP kinases [30]. The neurotrophin pathway has also been previously implied in immunity. In fact, neurotrophins and their receptors are key molecules in survival and functions of cells of both the innate and adaptive immune system [31]. This can possibly explain why combinations of TKI and immune checkpoint inhibitors work better as first line therapy for mRCC patients [32]. Furthermore, the tumor microenvironment (TME) has been suggested as an additional major player influencing on the effect and response of TKIs. Studies have shown that stromal cells in the TME exposed to TKIs produce cytokines, hormones, or growth factors that modulate the response of the tumor to TKIs [33, 34]. Interestingly, stromal cells can also secrete growth factors activating the MAPK and Pi3K/AKT/mTOR, leading to resistance of cancer cells [35, 36]. The role of TME in resistance to TKI treatment in RCC patients is however not fully explored and is an area that needs further investigations.

## MATERIALS AND METHODS

### Patient material

In total, 324 nephrectomy samples were consecutively collected at the Akershus University Hospital from 2013 to 2019. From these, we selected 25 RCC patients suitable for analysis as we had matching normal kidney tissue available. Twenty-two nephrectomy samples were available from patients with either primary or recurrent metastatic disease, and three with non-metastatic disease (see Table 3 for patient characteristics and Supplementary File 3 for extensive information). Twenty-three patients were classified with ccRCC histology, whereas one patient with chRCC, and one with pRCC. An experienced pathologist assessed tumor and normal tissue collected from each patient.

### Sample preparation

Tissue specimens from normal and cancer kidney tissue (*n* = 50) were sectioned with a cryostat (at –35°C) into 10 μm thick coupes, to a total volume of ∼15 mm^3^ for each sample. The number of coupes needed to get 15 mm^3^ for the experiment was calculated based on the surface area of the tissue specimen. Tissue samples were kept frozen at all times during the procedure, and to avoid contamination between tumor and normal tissue, all tumor tissue and normal tissue were sliced separately. The sectioned tissue was with 100–200 μl of mammalian protein extraction reagent (M-PER) buffer (Pierce Biotechnology, Inc., Rockford, IL), supplemented with phosphatase and protease inhibitors (Pierce Biotechnology, Inc.) The protein concentration of lysates was determined using the BCA assay (Pierce Biotechnology, Inc.).

### Tyrosine kinase activity profiling

PTK profiling was performed using the Protein Tyrosine Kinase PamChip^®^ Array for Pamstation^®^12 (PamGene International B.V., ‘s-Hertogenbosch, The Netherlands) at Akershus University Hospital, according to manufacturer’s protocol. Briefly, the assay contains 144 kinase substrates, representing approximately 100 different kinases. For each experimental run, 5 μg of protein sample lysate was added to the reaction mixture containing 1 × PK buffer, 10 mM DTT, 400 μM ATP, 1 × PTK additive (PamGene), 1:400 Halt Phosphatase Inhibitors (Thermo Fisher Scientific), 0.01% BSA (Pamgene), and fluorescein isothiocyanate-labeled antiphosphotyrosine antibody (PamGene), as previously described [27]. Based on pilot experiments of increasing concentrations of the different TKIs added *ex vivo* to kidney cancer tissue lysates, concentrations that resulted in ~50% inhibition of most kinase substrates were chosen for the main experiments. As a result, the PTK profiles of kidney cancer samples were assessed with and without the presence of four TKIs with following concentrations; 2.5 μM cabozantinib (XL184, BMS-907351) (Selleck Chemicals), 2.5 μM sunitinib (Sigma Aldrich), 10 μM pazopanib (Sigma Aldrich) and 2 μM tivozanib (AV-951) (Selleck Chemicals). Kidney cancer samples and normal tissue samples that were not treated with inhibitors (control) contained 2% Dimethyl Sulfoxide (DMSO) instead of TKIs. Initially, experiments were run in triplicates, but after confirmation of insignificant variation between technical replicates (coefficient of variation < 9%), most of our samples were not run in replicates to allow more individual samples to be used. However, for quality controls, some samples were run in duplicates or triplicates, where necessary.

### Data analysis

All data was processed and analyzed in Bionavigator v.6 (PamGene) interfaced to the statistical program R 3.3.1 (R-project, https://www.rproject.org/). Analysis performed included quality check, log2 transformation of data and averaging of the kinase signal replicates. During quality check, we excluded arrays that showed clear defects (broken array etc.). Signal-positive spots had to show a positive trend in the phosphorylation time course. Substrates in which we could not detect a positive trend in >75% of the samples were excluded from further analysis, resulting in 104/144 kinase substrates eligible for further analysis. For samples in which we had several replicates, the values from each replicate were averaged to contain only a single value per sample per kinase substrate. To identify significant differences in kinase activity between groups, student’s *T*-test or Mann–Whitney *U* test was used wherever appropriate to obtain *P*-values and false discovery rate (FDR). False discovery rate (FDR) < 0.05 was considered significant.

### Upstream kinase analysis

BioNavigator software v.6 (PamGene Inc.) was used to perform upstream kinase analysis in order to identify kinases that might be responsible for the differences in kinase activity observed between two groups, normal and cancer tissue. The method uses *in silico* predictions to identify upstream kinases through Kinexus Kinase Predictor (http://www.phosphonet.ca/), as previously described [37]. The method calculates a significance score or specificity score of a kinase Q = −10log [max (m/M, 1/M)], where m is the number of times out of M permutations that | τp | >| τ |, where τp is the value of the statistical difference obtained after permutation of the sample or peptide labels, respectively. Kinases are then ranked based on the sum of both scores. The kinases that rank on top, are the ones that are most likely to drive the differences between two groups.

### Pathway analysis

For kinase substrates that were significantly different between normal and cancer tissue, pathway analysis was performed using the UNIPROT accession number for each kinase substrate involved, using DAVIDS bioinformatics [38] and reactome (https://reactome.org/cite) online software tools.

## CONCLUSIONS

The results of our study contribute to better understanding of the changes in kinase activity in RCC tumor cells involved in fundamental oncogenic cellular processes and the *ex vivo* effect of TKIs. We found tivozanib and cabozantinib to be more potent TKIs in RCC samples than sunitinib or pazopanib. The next step will be to correlate the efficacy and toxicity in individual patients with their respective kinase activity of normal and malignant kidney tissue. Thus, the presented findings might provide options to select the most promising TKI for individual RCC patients prior to initiation of TKI-therapies.

## SUPPLEMENTARY MATERIALS















## Abbreviations

ccRCC: clear cell renal cell carcinoma
ChRCC: Chromophobe Renal Carcinoma
chRCC: chromophobe renal cell carcinoma
DMSO: Dimethyl Sulfoxide
FDR: false discovery rate
LFC: log fold change
mRCC: metastatic renal cell carcinoma
mTOR: mammalian target of rapamycin
PDGFR: platelet derived growth factor receptor
PI3K: Phosphoinositide 3- Kinase
pRCC: papillary Renal Carcinoma
pRCC: papillary renal cell carcinoma
PTK: protein tyrosine kinase
RCC: Renal cell carcinoma
sRCC: renal cell carcinoma with sarcomatoid features
TKI: Tyrosine kinase inhibitor
TME: tumor microenvironment
VEFR: vascular endothelial growth factor receptor
VEGF: vascular endothelial growth factor
