# Supplementary material for: Kinase activity profiling in renal cell carcinoma, benign renal tissue and in response to four different tyrosine kinase inhibitors
Source: Oncotarget. 2022 Aug 4;13:970–81. doi: 10.18632/oncotarget.28257 (PMC9450987; doi:10.18632/oncotarget.28257)
Supplement: Supplementary file 1 [file oncotarget-13-28257-s001.pdf]

Kinase activity profiling in renal cell carcinoma, benign renal tissue and response to four different tyrosine kinase inhibitors

SUPPLEMENTARY MATERIALS

Supplementary File 1: Table including all kinase substrates with *p*.values and log fold change (LFC) between untreated (control) and treated samples with each TKI, and which cluster each kinase substrate is part of. See Supplementary File 1

Supplementary File 2: Kinase substrates and pathway analysis in cluster 2 and 3. See Supplementary File 2

Supplementary File 3: Extended clinical information for each patient. See Supplementary File 3

Normal_tumor_dataset1 : Tyrosine kinase substrate log2 values of cancer and normal kidney tissue. See Supplementary Dataset 1

Control and compound_dataset2: Tyrosine kinase substrate log2 values **of cancer tissue treated with four different tyrosine kinase inhibitors. Ctrl: cancer samples treated with DMSO. Tivo:tivozanib, cabo: cabozantinib, sut: sunitinib.** See Supplementary Dataset 2

Enrichmentfile.dataset.3: Enrichment file for the datasets with information for each sample used for the different tyrosine kinase profiling runs. See Supplementary Dataset 3
